# Prediction and preliminary validation of oncogene regulation by miRNAs

**DOI:** 10.1186/1471-2199-8-79

**Published:** 2007-09-18

**Authors:** Edyta Koscianska, Vesselin Baev, Konstantinia Skreka, Katerina Oikonomaki, Ventsislav Rusinov, Martin Tabler, Kriton Kalantidis

**Affiliations:** 1 Institute of Molecular Biology and Biotechnology, Foundation for Research and Technology, Hellas, PO Box 1385, GR-71110, Heraklion/Crete, Greece; 2 Department of Plant Physiology and Molecular Biology, University of Plovdiv 24, Tsar Assen St, 4000 Plovdiv, Bulgaria; 3 Department of Biology, University of Crete, Heraklion, Greece; 4 Laboratory of Cancer Genetics, Institute of Bioorganic Chemistry, Polish Academy of Sciences, Noskowskiego 12/14, 61-704 Poznan, Poland

## Abstract

**Background:**

MicroRNAs (miRNAs) are one of the most abundant groups of regulatory genes in multicellular organisms, playing important roles in many fundamental cellular processes. More than four hundred miRNAs have been identified in humans and the deregulation of miRNA expression has been also shown in many cancers. Despite the postulated involvement of miRNAs in tumourigenesis, there are only a few examples where an oncogene or a tumour suppressor has been identified as a miRNA target.

**Results:**

Here, we present an *in silico *analysis of potential miRNA- oncogene interactions. Moreover, we have tested the validity of two possible interactions of miRNAs with genes related to cancer. We present evidence for the down-regulation of *c-MYC*, one of the most potent and frequently deregulated oncogenes, by let-7 miRNA, via the predicted binding site in the 3'UTR, and verify the suppression of *BCL-2 *by miR16.

**Conclusion:**

In this work both bioinformatic and experimental approaches for the prediction and validation of possible targets for miRNAs have been used. A list of putative targets for different oncomirs, validation of which would be of special interest, is proposed and two such interactions have been experimentally validated.

## Background

Originally discovered as a rare example of single-stranded ~22-nt RNA regulators of developmental timing in *C. elegans*, microRNAs are now regarded as playing an important regulatory role in gene expression of animals and plants [1-5]. Even more, it has turned out that miRNAs are one of the most abundant groups of regulatory genes in multicellular organisms [6]. These small RNAs negatively regulate gene expression at the post-transcriptional level involving duplex formation between the miRNA and the messenger RNA. It is believed that the level of complementarity between these two molecules is decisive of the type of regulation achieved by the miRNAs. When the binding is supported by extensive or complete complementarity to the coding region of the mRNA, the RNAi pathway is induced, resulting in the cleavage and degradation of the target transcript. This type of regulation, involving RNA-induced silencing complex (miRISC) and miRNA-directed mRNA cleavage, is widely observed in plants [5,7-9] but has been also reported to occur in mammals [10]. In animals, however, miRNA-mRNA binding is more variable, it may contain several mismatches, loops and bulges, and it is found to take place mainly in the 3'UTR of the messenger molecule [11-16]. This imperfect complementarity leads to another type of gene regulation which does not include mRNA cleavage but apparently inhibition of translation [17,18]. In this case the protein levels are reduced, but the mRNA abundance in the cell remains unchanged. Besides these two types of regulation, recent studies have indicated that there is at least an additional way in which miRNAs can negatively influence gene expression. By the same incomplete pairing with the 3'UTR, miRNAs are able to destabilize and reduce mRNA concentration, by accelerating poly (A) tail removal leading to miRNA-mediated mRNA decay [19,20]. This type of mechanism is irreversible, like the mRNA cleavage, and seems to be an important part of the miRNA-regulatory network. Another significant feature of the microRNA binding is the crucial pairing of the miRNA 5'end nucleotides [21-25]. Brennecke et al. [26] have suggested the general structural requirements for the functionality of miRNA sites with the incomplete complementarity.

Since the discovery of miRNAs, their number and the examples of their possible functions have dramatically grown. It has been shown that the expression levels of particular miRNAs differ depending on the developmental stage, cell type and tissue [27-29]. Altered expression pattern (over- or under-expression) of specific miRNAs has been also reported in tissues derived from various tumours [3,30-33]. A very large portion (more than 50%) of the human miRNAs are located in so-called "fragile sites", on chromosomes which are frequently deleted, amplified or rearranged in case of cancer development [34]. This data together suggest that the aberrant activity of miRNAs, influencing basic cellular processes such as growth, proliferation and differentiation of cells, may contribute to tumourigenesis. The possible functions of cancer-related microRNAs, termed recently "oncomirs" [31,35], have been therefore widely investigated (reviewed in [35-38]). It was found that miR-15a and miR-16 were deleted or downregulated in lymphocytic leukaemia [39]; let-7 was downregulated in lung cancers [40,41]; the miR-17 cluster was amplified in several types of lymphoma and solid tumours [31,42,43]; miR-21 was overexpressed in glioblastoma [44,45] and breast cancer [46]; levels of miR-143 and miR-145 were decreased in colorectal neoplasia, breast, prostate and cervical cancers [46,47]; miR155 was upregulated in Burkitt and B-cell lymphomas [48-50] and also in breast cancer [46]. Although deregulation of expression of many miRNAs has been already reported in different cancers, the direct interaction between the particular miRNA and mRNA of a specific oncogene has been proved only in a few cases. More specifically, experimentally has been shown, the suppression of *RAS *oncogene by let-7 [40]; the suppression of *BCL-2 *by miR-15a and miR-1 [51]; the regulation of transcription factor *E2F1 *activity by miR-17-5p and miR-20 [52]; the downregulation of the *KIT *oncogene by miR-221 and miR-222 [53], the inhibition of the expression of tumour-supressor *LATS2 *and the influence on p53 pathway by miR-372 and miR-373 [54], and finally, the downregulation of the proto-oncogene *BCL6 *by miR-127 [55].

In this work, we present both computational and experimental approaches for verification of human miRNA target sites. Using bioinformatics methods, we have found a large number of potential target sites for miRNAs in the group of oncogenes and genes related to cancer. Further, by an experimental approach, we have tested the validity of two possible interactions. We present here evidence that let-7 binds to the 3'UTR of *c-MYC *oncogene and downregulates its expression. Moreover, we have confirmed that miR-16 regulates *BCL-2*, (previously reported by others [51]), and we have proposed the other putative targets for different oncomirs, validation of which would be of special interest.

## Results

### *In silico *analysis

The goal of the bioinformatic analysis was to find potential binding sites for cloned miRNAs in the set of human oncogenes. In humans, as in other animals, the miRNA regulation is accomplished by binding the microRNAs predominantly to the 3' untranslated region (UTR) of the messenger RNA. [21,56,57]. For this reason the investigation and scanning for miRNA:mRNA binding sites was limited to 3'UTR sequences of *Homo sapiens*.

After the scanning and filtering of the UTRs oncogenes dataset (149 sequences), positive results for 294 binding sites in 80 sequences (genes) from this set were obtained. Table 1 presents an arbitrary selection of known oncogenes with their predicted miR-regulators as examples of the analysis. The whole list is placed as supplementary material (Additional file 1). Most of the oncogenes presented contain multiple predicted binding sites in their UTRs, which is in accordance with the current understanding that a gene is usually regulated by more than one miRNA, or else contains multiple sites of one miRNA for a combinatorial or more efficient regulation [18,58,59].

**Table 1:** Examples of predicted miRNA/oncogene interactions

**gene name**	**3'UTR position**	**miRNA name**	**secondary structure**	**free energy**
*BCL-2*	1200	hsa-miR-139	((((((((.(((((((((&)))))))))..)))))))).............	-27.5
*BCL-2*	2382	hsa-miR-370	((((...((.(((((((((((&))))))))))).)).)))).............	-36.5
*BCL-2*	2500	hsa-miR-16	..(((((.....((((((((((&))))))))))..)))))...............	-23.3
*BCL-2*	2500	hsa-miR-195	.(((((....(((((((((((&))))))))))).)))))...............	-26.5

*c-MYC*	114	hsa-let-7c	(((((((......(((((((((&)))))))))......)))))))..........	-24.5
*c-MYC*	114	hsa-let-7b	(((((((......(((((((((&)))))))))......)))))))..........	-24.5

*ERB B2*	37	hsa-miR-214	..(((((.((...(((((((.&.))))))))).)))))................	-24.6
*ERB B2*	361	hsa-miR-132	(((((........(((((((..&..))))))).......)))))...........	-23.26

*ERB B3*	9	hsa-miR-302d	.((((((.....(((((((((((&)))))))))))))))))...............	-25.00
*ERB B3*	254	hsa-miR-515-3p	..(((((......((((((((&)))))))).....)))))..............	-22.60
*ERB B3*	648	hsa-miR-17-5p	..((((((((.....(((((((((&))))))))).....))))))))..........	-26.80
*ERB B3*	648	hsa-miR-20b	.((((((((.....(((((((((&))))))))).....))))))))..........	-26.80
*ERB B3*	648	hsa-miR-20a	.((((((((.....(((((((((&))))))))).....))))))))..........	-25.50
*ERB B3*	648	hsa-miR-106a	..((((((((.....((((((((.&.)))))))).....))))))))..........	-25.10

*FRAT 1*	127	hsa-miR-125b	((((((((....(((((((((.&..))).))))))))))))))............	-23.8
*FRAT 1*	127	hsa-miR-100	(((((((.....((.((((((.&.))))))))...))))))).............	-24.7
*FRAT 1*	271	hsa-let-7b	(((((((.(((((((..(((((&)))))....))))))).)))))))........	-30.8
*FRAT 1*	796	hsa-miR-34a	.(((((((((......((((((&)))))).)))))))))................	-23.4
*FRAT 1*	1295	hsa-miR-15b	((((((((((((((...((((.&))))...))))))))))))))...........	-27.6

*K-RAS*	2004	hsa-miR-371	.((((....((((((((((..&.)))))))))).......))))..........	-24.8
*K-RAS*	2294	hsa-miR-92	.((((((((......(((((((&)))))))..))))))))...............	-23.4
*K-RAS*	3137	hsa-miR-147	.(((...((((((((((((.&..)))))))))))).....)))..........	-23.5
*K-RAS*	4832	hsa-miR-125a	.((((((.....(((((((((..&.))))))))).....))))))...........	-26.6
*K-RAS*	4946	hsa-miR-147	.((((((((..(((((((((&.))))))))).....)))))))).........	-24.3

*K-RAS*	2355	hsa-let-7i	..((((....((..(((((((&)))))))..))...))))..............	-18.8
*K-RAS*	2783	hsa-let-7f	((((...(((((.(((((((((&)))))))))..)))))...)))).........	-18.3
*K-RAS*	3125	hsa-let-7b	((((((((((.....(((((((&)))))))))))))..)))).............	-18.6
*K-RAS*	4919	hsa-let-7b	(..(((.((((..(((((((((&)))))))))..)))))))..)...........	-24.8
*K-RAS*	4919	hsa-let-7c	..(((..((((..(((((((((&)))))))))..)))).....))).........	-21.4
*K-RAS*	4919	hsa-let-7i	.((((((.(((.(((((((((&)))))))))..))).))).)))..........	-19.6

Examples of predicted miRNA/oncogene interactions. Several important oncogenes and their potential miRNA regulators were arbitrary selected from the full list of 294 genes (Additional file 1), to be shown as examples. RNAfold "dot-and-bracket" format was used to show secondary structures of miRNA/mRNA duplexes (left side of appropriate structure corresponds to miRNA sequence in 3'-5'orientation; right side corresponds to predicted mRNA binding site in 5'-3' orientation). Brackets represent paired and dots non-paired nucleotides, respectively.

From the unfiltered results obtained automatically by the programme, we chose the group of records showing relatively weak 3' pairing but very strong and extensive 5' pairing (seed site), which is characteristic of strong and potentially positive targets [59].

**Figure 1:**
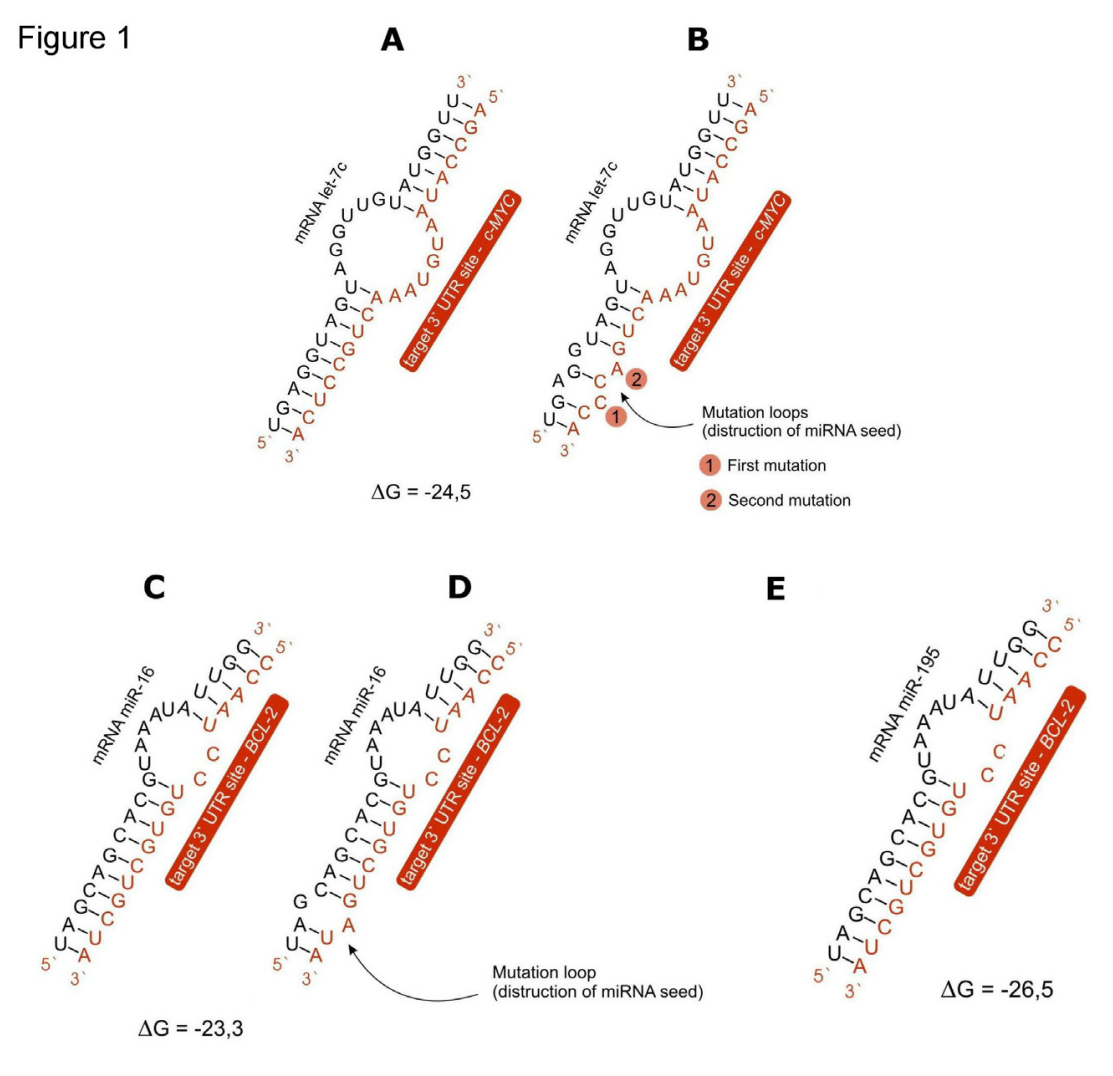
**Schematic representation of the predicted interaction between miRNA and the mRNA target 3'UTR site**. Figures (A) and (C) show wild-type interactions for *c-MYC*/7c and *BCL-2*/16, respectively. In figures (B) and (D) the predicted interaction between miRNA and the respective mutated binding site is depicted (B for *c-MYC*, D for *BCL-2 *). (E) microRNA 195 from the miR-15 family was predicted to be a reliable regulator for *BCL-2 *gene (see text for more details).

Of the interactions predicted, we thought that the potential interaction between the oncogene *c-MYC *and the let-7c miRNA could be of particular interest due to the known importance of the gene and the conservation of the specific miRNA. The secondary structure of this pair shows good complementarity in the 5' of the let-7c miRNA (9 nt) which is considered very important for the target recognition [56]. In addition, good pairing in the 3' region was found *in silico *(Fig. 1A). The duplex structure seems to have all the properties for an animal miRNA binding that are needed for down-regulation of a gene. The binding of let-7c miRNA is in position 114 of the 3'UTR. Furthermore, independently of the first analysis, we inspected in detail miRNAs binding sites of human and murine forms of the *MYC *gene. In each tested *MYC *mRNA we could detect and identify in the 3' UTR a binding site for a representative of the human/murine let-7 member of miRNAs (Additional file 2).

Sethupathy et al. [60] analysed human/murine experimentally validated examples and showed that there is a tendency for the binding sites to occur closer to the start of the UTR than to the end. We have also checked all the human/murine *MYC *potential interactions with let-7 and found that they are all located in the 2/3-th region of the 3'UTR. However, there is no obligatory region that has been proven so far for the localization of the miRNA binding sites within the 3' UTR. miRNA sites also have preferences to the of 3'UTRs sequence composition. According to Robins et al., the large majority (~75%) of human miRNA target genes have AT-rich 3'UTRs [61]. We have tested our human/murine genes, and the great majority have high AT content (between 51% and 70%, with an average of 61%).

Our *in silico *analysis revealed also an additional interaction of potential interest (Fig. 1C). It has been previously reported that miR-15a and miR-16-1 target *BCL2 *mRNA [51]. We have found that also another member of the miRNA 15 family is predicted to bind exactly on the same site as miR-16 (pos. 2500 in the *BCL2 *3'UTR). This miRNA, miR-195, shares the same seed region with miR-15 and miR-16 and shows high similarity to the rest of the miR-15 family sequence. The free energy of the miR-195/*BCL2 *(26.5 kcal/mol) is even stronger than for miR-16/*BCL2 *(23.3 kcal/mol). The secondary structure is the same perfectly symmetrical 5'dominant site, with an even smaller central loop (Fig. 1E). The microarray analysis revealed that miR-195 is an immuno-specific miRNA, and is expressed at a high level in all organs of the immune system [62]. This miRNA is abundant in lymphocytes and is encoded in a region of chromosome 17 which is missing in some forms of CLL [63-65].

### Experimental validation

We concentrated on target genes forming almost perfect duplexes with their miRNA partners in the 5' "seed" region, with a central loop and having an arbitrary free energy around -23 kcal/mol, which is a typical energy value for formation of this sort of structures. Therefore, two very important oncogenes *BCL-2 *and *c-MYC *were chosen from the list for further investigation, and their potential micro-regulators miR-16 and let-7c, respectively.

**Figure 2:**
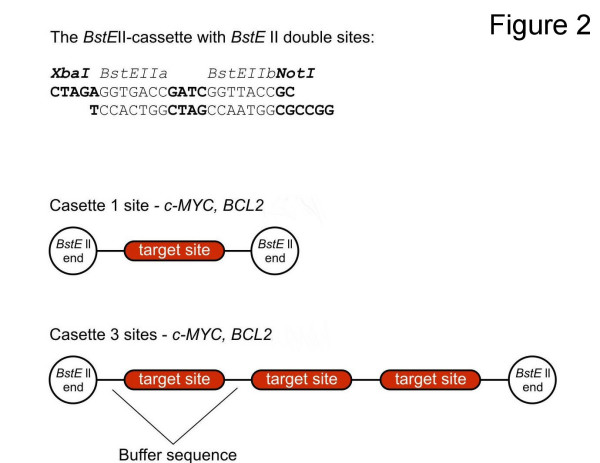
**Constructs used for the validation of miRNA- oncogene interaction**. The sequence of the *Bst*EII cassette is shown on the top. Predicted binding sites for appropriate miRNAs were inserted into *Bst*EII site. Single target sites or triplets were used for *c-MYC *and *BCL-2 *genes (for sequences see the Additional file 4).

For the experimental verification of the predicted interactions between selected miRNAs and their putative targets, experiments using sensor constructs carrying a *Renilla *luciferase reporter were performed. miRNAs' activity on reporter genes bearing the miRNA-binding sites have been previously demonstrated [22,23,66]. Only sequences carrying binding sites for appropriate miRNAs were used. Since it is known that most of the mammalian targets often contain binding sites for multiple miRNA [23], constructs carrying a single binding site for the tested miRNA and constructs carrying binding sites repeated three times were used (Fig. 2). Moreover, constructs having mutations disrupting the native pairing within the binding region (5' seed site) of the candidate miRNAs (designated as MUT) and constructs showing perfect complementarity (PM) to them were generated as well, in order to provide a negative and a positive control, respectively. All types of cassettes prepared were placed into the phRL-TK-*Bst*EII vector, downstream of the hR*luc *gene at *Bst*EII sites.

We performed the transfection of HeLa cells for sensors of *BCL-2 *and *c-MYC *miRNA potential binding sites. Each time, four constructs were tested in parallel: an empty luciferase vector (Control), wt potential binding site for appropriate miRNA (WT_b.s), mutated binding site (MUT_b.s) and site with full complementarity (PM_b.s). Since the miRNAs of interest have been shown to be expressed in HeLa cells at relatively high levels [62] there was no need for miRNA precursor over-expression. Luciferase activity was measured and normalised against the β-galactosidase expression.

**Figure 3:**
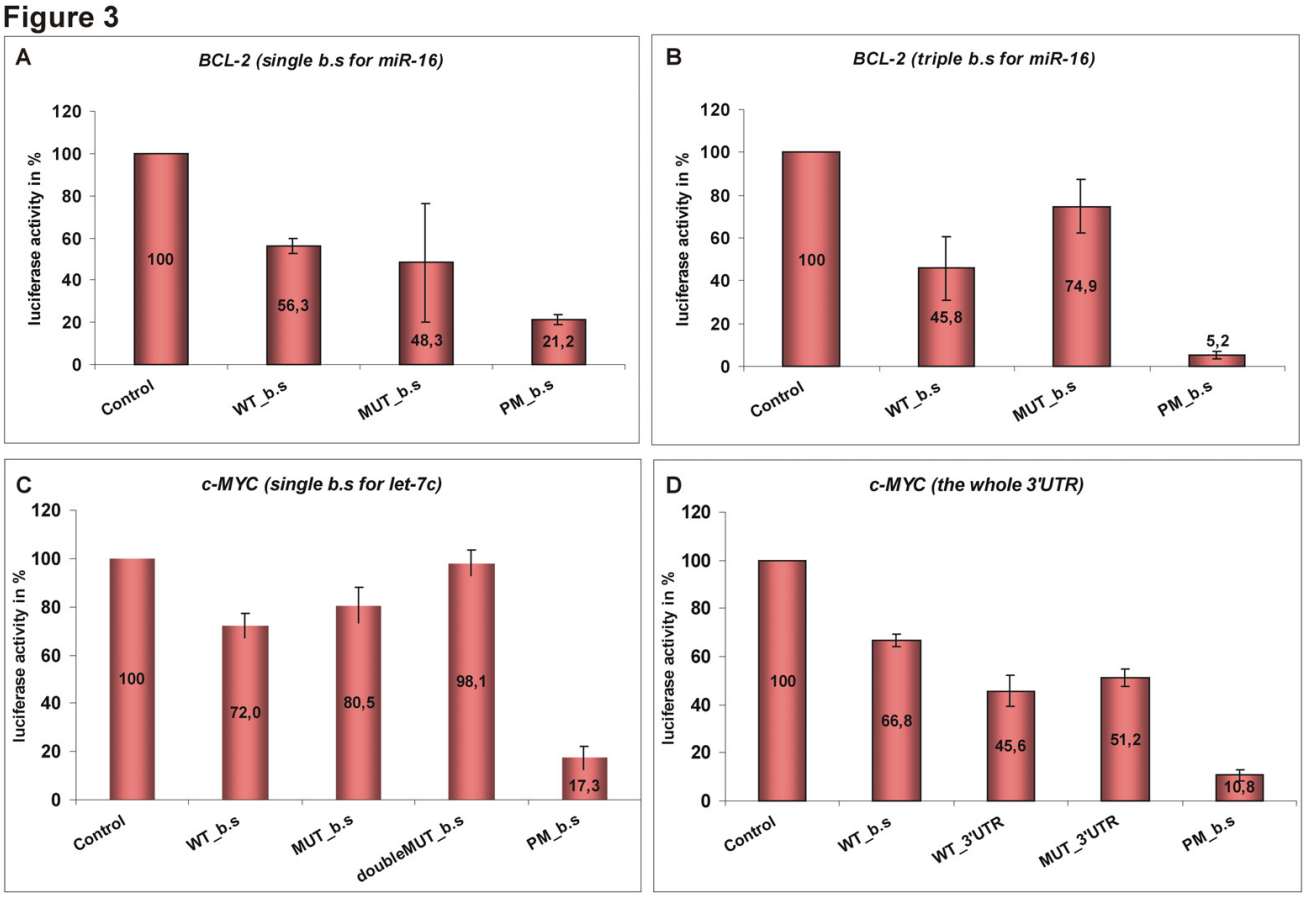
**Relative repression of luciferase expression standardised to a transfection control**. The following reporter constructs were tested: (A) *BCL-2 *reporter constructs carrying a single binding site for miR-16, (B) *BCL-2 *reporter construct carrying a triple binding site for miR-16, (C) *c-MYC *reporter constructs carrying a single binding site for let-7, (D) *c-MYC *reporter constructs including sensors carrying the whole 3'UTRs from the *c-MYC *gene. The luciferase activity was normalised against *β-gal *expression. Results are presented in % (100% – luciferase vector with no binding site, (+) control). Average from several experiments is shown (details in the text).

Although at the time we were conducting our experiments the regulation of *BCL-2 *by miR-16 was reported [51], we nevertheless decided to include this interaction in the study, as an additional control of the approach we used for miRNA target verification. In our system, using only the expected miR-16 binding site of *BCL-2 *3'UTR, we confirmed that *BCL-2 *oncogene is targeted by miR-16. More specifically, we obtained 1.78 fold reduction of the luciferase activity even when the sensor construct carried a single binding site for miR-16. The activity was decreased up to 56% compared to 100% in the control (Fig. 3A). The suppression was stronger when a construct that contained a triple miR-16 binding site was used and resulted in 2.18 fold downregulation – only 45.8% of the original luciferase activity measured (Fig. 3B). The HeLa transfections were repeated 6 and 10 times, respectively, and the score is presented as an average.

The sensor construct carrying the *c-MYC *potential single binding site for let-7c appeared to be consistently well down-regulated by let-7 microRNA. The luciferase activity dropped to 72% of the empty vector (about 1.41 fold reduction). Since a single mutation at position 3 of the "seed" in the *c-MYC *MUT_b.s -sensor (our negative control) – was not sufficient for full reversion of the luciferase activity compared to WT_b.s, we decided to test also *c-MYC *doubleMUT_b.s, a construct bearing two mutations in the 5' "seed" element, one at position 3 and one at position 5 (Fig. 1B). The transfection experiments confirmed that the down-regulation of *c-MYC *oncogene is highly reproducible. As expected, the luciferase activity for *c-MYC *doubleMUT_b.s showed a more efficient de-repression, becoming almost equal to the control experiment (98.1%). Conversely, the activity of luciferase measured for the *c-MYC *PM_b.s construct was very low (17.3%). The data represent the average score from 10 independent experiments (Fig. 3C).

To confirm further that *cMYC *oncogene is a real target for let-7c we performed next transfections of HeLa cells, using anti-let-7c inhibitor in order to block the predicted interaction of let-7c with our sensor constructs. The co-transfection of anti let-7 resulted in derepression of the luciferase activity in sensor constructs (the exception was the construct bearing PM b.s for *c-MYC*), although derepression was not equally efficient for all sensor constructs (Additional file 3).

It is worth noting, that all perfect matching constructs used as positive controls interacting with the miRNA under investigation through the siRNA pathway [10,67,68] worked consistently well. The luciferase activity in all studied cases was always repressed at a very low level, ranging from 5% up to 30% of the empty reporter construct.

Further we intended to investigate whether the effect of let-7 on the *c-MYC *binding site-containing reporter could be recapitulated when the whole 3'UTR of *c-MYC *was present downstream of the luciferase gene. This is important since it is possible that structural features of the UTR may hinder the miRNA/target interaction. For this purpose, the whole *c-MYC *3'UTR was cloned into the luciferase vector. In addition, two point mutations in the seed region of the potential let-7c b.s in the *c-MYC *3'UTR were introduced, similarly to the *c-MYC *doubleMUT_b.s sensor construct carrying only the let-7c b.s (compare to Fig. 1B). Again, a series of HeLa transfections was performed. Five constructs were tested in parallel: an empty luciferase vector (negative control), the construct carrying the potential b.s for let-7c (WT_b.s), the construct with the whole *cMYC *3'UTR (WT_3'UTR), the construct with the mutated *c-MYC *3'UTR (MUT_3'UTR) and the construct carrying b.s with full complementarity (PM_b.s – our positive control). Both *c-MYC *constructs, the one carrying the potential let-7c binding site only and the construct carrying the whole 3'UTR of c-*MYC*, showed a suppression of the luciferase activity compared to the control vector, although not at equal levels to each other. The construct with the single let-7 b.s reduced the luciferase activity to 66.8 % of the control, while for the construct with the whole 3'UTR the reduction was on average up to 45.6 %. In the case of the *c-MYC *MUT_3'UTR sensor the luciferase activity reached 51.2 % of the control, suggesting that the mutation disturbing the potential b.s for let-7c restored the activity of luciferase when compared to the WT_3'UTR sensor, however, not efficiently compared with the construct bearing the let-7 binding site only (WT_b.s). The HeLa transfections and luciferase assays were repeated 6 times and the score is presented as an average (Fig. 3D).

**Figure 4:**
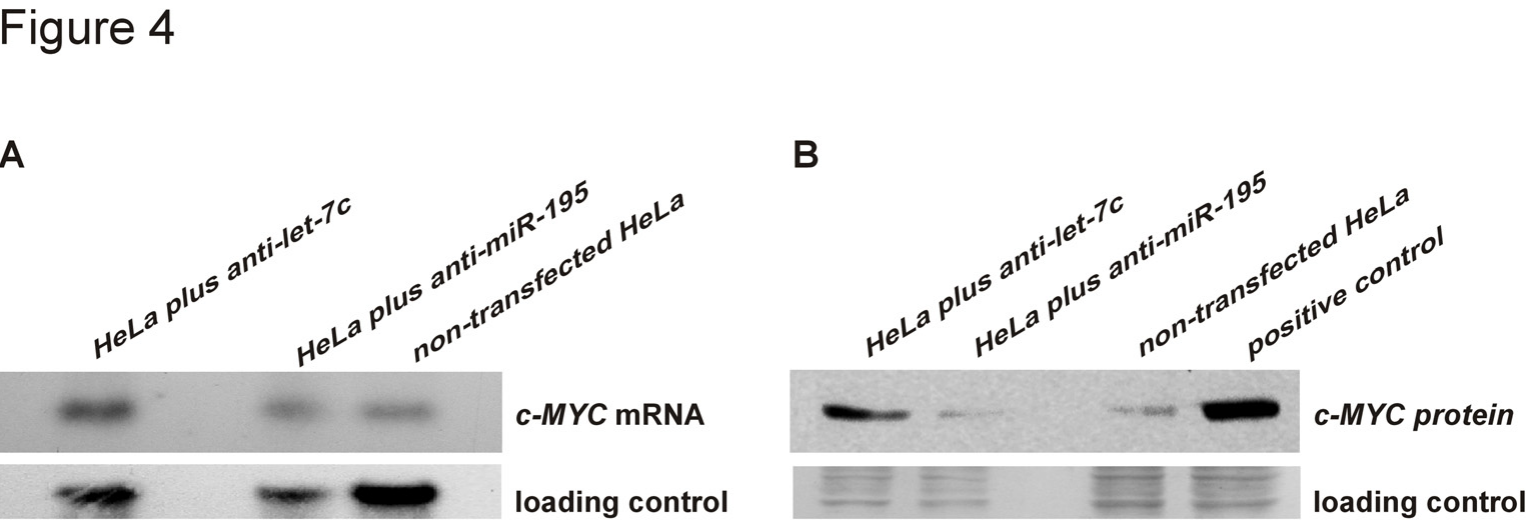
**Inhibition of let-7c enhances *cMYC *mRNA and protein levels**. Northern blot (A) and Western blot (B) and analyses were performed 48 hours after transfection of HeLa cells with anti-let-7c inhibitor. RNAs and protein samples were extracted from non-transfected HeLa cells and cells transfected with the anti-let-7c and anti-miR-195 oligos, as indicated in figures. In case of the western analysis a positive control (protein extract from lymphoma cells overexpressing cMYC) was included as well.

Finally, we determined the extent to which endogenously-expressed *c-MYC *is subjected to the regulation by let-7 microRNA. We have monitored *c-MYC *expression at the mRNA and protein levels following the inhibition of let-7 with the antisense synthetic let-7 oligo (anti-let-7c from Dharmacon) in HeLa cells. 48 h post-transfection RNAs and proteins were extracted from HeLa cells and analysed by Northern and Western methods, respectively. We detected a mild increase at the mRNA level and quite a significant increase at the protein level (Fig. 4A and 4B). In addition to the increase of c-MYC protein level, we also observed accelerated growth rate of the transfected HeLa cells, eventually causing early cells' death (not shown). On the contrary to the specific effect caused by the introduction of anti-let-7c, we did not observe similar changes, either at mRNA or protein levels, when an irrelevant anti-miR was used (synthetic antisense *miR-195 *oligo).

## Discussion

More than 400 human miRNAs have been identified so far [69] and bioinformatics analyses predict that the total number may be around 1000 [6,70]. More than 200 miRNAs have been already correlated with cancer [30]. Moreover, the expression profile of miRNAs from cancer tissues has been suggested to be prognostic concerning the speed of cancer development and its malignancy [41,71,72]. A correlation between the expression of particular miRNAs and their effects on target oncogenes followed by tumourigenesis is beginning to gain evidence. Of particular interest is the possibility that the growth of cancer cells can be repressed by artificial modulation of the level of miRNAs [73-75]. Despite the accelerated research interest in the miRNA-regulation, there are only a few examples where an oncogene or a tumour suppressor has been identified as a miRNA target and the regulatory connection validated [40,51,53,54]. Our results indicate that the expression of the *c-MYC *gene, which is one of the critical oncogenes, is modulated by let-7, expanding the number of validated oncomirs.

Since the significant increase of miRNA targets, all previous validations (including some "oncomirs") have been gathered by Sethupathy at al, in a database called TarBase [60]. The assay of luciferase as a reporter gene has been widely used for the verification of human targets; more than 80% of human targets in the TarBase were validated by this method. However, this validation system has variations depending on the inserted fragment of the target 3'UTR. Either the full-length 3'UTR of mRNA [40,53,76], or only a segment containing the binding site [23,51], or even only the miRNA predicted binding site [22,52] has been inserted in appropriate reporter constructs. Insertion of multiple binding sites should ensure higher efficiency of the sensor used, since cooperativity of multiple miRNA binding sites has been reported [77,78]. However, the degree of miRNA-mediated translational repression may depend also on different factors, for example particular miRNA and mRNA concentrations, the accessibility of the binding site and its context [22]. Moreover, the validations mentioned above were achieved by different experimental techniques and for different miRNAs, making it difficult to compare the results.

In the present work we have used synthetically designed miRNA-binding sites for miRNAs of interest, cloned downstream of the luciferase reporter gene. We have tested constructs with single and triple binding sites. We were somewhat surprised to find that a considerable down-regulation was achieved even when sensors carried only one binding site, without the addition of miRNA precursors. A significant repression was observed for both *c-MYC *and *BCL-2 *single binding sites. The miRNAs studied here, let-7c and miR-16, are predicted to interact with their potential binding sites *c-MYC *and *BCL-2 *respectively, in a so-called canonical interaction, in agreement with the report that the canonical sites have higher binding energy and may be more efficient suppressors [26]. However, the use of triplet constructs not only resulted in the stronger regulation but also ensured more reproducible outcome. Moreover, we could show that the predicted miRNA binding site functions in the context of the entire 3'UTR. Cloning of the whole *c-MYC *3'UTR into the luciferase vector resulted in a very strong reduction of the luciferase activity, the strongest effect observed in our set of experiments. Mutagenesis of two nucleotides in the let-7c binding site (nt 3 and 5 in the seed region) could only marginally restore the luciferase activity, although the same mutations caused significant upregulation of luciferase expression in the b.s construct (Fig 3D). This indicates that other miRNA binding sites present in the *c-MYC *3'UTR cooperate in the modulation of gene expression. Indeed, other miRNA binding sites have been bioinformatically identified in the *c-MYC *3'UTR (not shown). We believe that the interaction studied here, let-7c/*c-MYC *(pos. 114 of the UTR) is the strongest and the most important for the regulation of *c-MYC *expression, however, miRNAs are likely to contribute to the regulation of *cMYC*.

Although now there are many programmes established for the computational prediction of miRNA targets (reviewed in [79]), the large number of predicted targets makes it difficult to choose the best candidates for further experimental verification in a biological system. The advantage of our bioinformatics approach is the possibility of relatively fast, manual scanning of the secondary structures formed by analysed pairs of putative interactors through the use of the RNAfold dot-and-bracket format. It is worth noticing, that in the results list of our scanning important oncogenes can be found (for example *BCL-2 *and *RAS*), that have already been proven as targets for particular miRNA (see Additional file 1). Such an outcome supports the suitability of the scanning algorithm used here.

So far, only a handful of miRNAs and their targets have been characterized in detail. Although the *BCL-2*/miR-16 interaction has been already shown [51], we demonstrate it here with the use of *BCL-2 *binding site for miR-16 only and not *BCL-2 *3'UTR segment of 546 bp, as before [51]. Here we demonstrate that *c-MYC *is targeted by let-7. We show that 22-nt sequence from the *c-MYC *3'UTR, predicted to be a binding site for let-7c, is enough to cause down-regulation of a reporter gene in HeLa cells. In addition, suppression of let-7 miRNAs in these cells by anti-let-7 significantly recovers reporter gene expression. Moreover, the suppression of let-7 in HeLa affects also the expression of the endogenous *c-MYC*, leading to an increase at both mRNA and protein levels. Mammalian miRNAs affecting target mRNA levels in addition to protein levels have been reported before [40,80] although there are also examples of endogenous mammalian mRNAs that are regulated by miRNAs at the level of translation without any change in target mRNA levels [51,55].

Based on our analysis it is not possible to specify which member(s) of the let-7 family is (are) responsible for the down-regulation of *c-MYC*. Let-7c, let-7b and let-7i (all with theoretically good efficiency) may bind to the same site in 3'UTR of *c-MYC *(Table 2). Biological data may give some clues as to which of the let-7 family members may be more likely to interact with *c-MYC *in vivo. In 80% of breast cancers, *c-MYC *is over-expressed, indicating that its potential miRNA regulator could be down-regulated in these cases. However, the expression level of let-7b both in normal and cancer breast tissues is the same [32]. Moreover, the down-regulation of let-7 family members (including let-7c but excluding let-7b) has been reported elsewhere for breast cancer [46]. In this last report, the down-regulation of let-7 was shown to correlate with metastasis or higher proliferation index, which could additionally support our hypothesis for *c-MYC *involvement [46]. Furthermore, the next evidence for our prediction of *c-MYC*/let-7c interaction is the fact that frequent deletions in chromosome 21 (21q21) have been reported in cases of lung and colorectal carcinomas and their metastases [81-84]. The same genomic location is shared by the let-7c gene, while the other candidates such as let-7b or 7i are located on chromosomes 12 and 22, respectively. The deletions in a region coding let-7c would be the simplest explanation for the reduced level of let-7c in cancer tissues, the miRNA diminishing the activity of *c-MYC*, therefore contributing to the enhanced proliferation and tumourigenesis.

**Table 2:** Possible let-7 family members that can interact with 3'UTR of *c-MYC*

**3'UTR position**	**miRNA name**	**secondary structure**	**free energy**
114	hsa-let-7i	.(((((...(((((((((&))))))))).....)))).)))).........	-20,5
114	hsa-let-7b	(((((((......(((((((((&)))))))))......))))))..........	-24,5
114	hsa-let-7c	(((((((......(((((((((&)))))))))......))))))..........	-24,5
114	hsa-let-98	.(((((((((...(((((((((&))))))))))))))))))..............	-20,8

It has been shown that the concentration of the MYC protein is critical for the conversion, persistence or regression of the liver tumours [85]. The cause of spontaneous *c-MYC *up-regulation is not yet clear. Calin *at al*. proposed that chromosomal translocation could bring *MYC *genes under the control of promoters for microRNAs, so that the gene will be over-expressed [39]. An alternative trigger for *c-MYC *over-expression could be reduced activity of the regulator, such as a let-7c that negatively modulates gene expression at translational level. Spontaneous *c-MYC *over-expression could be therefore the result of down-regulation or loss of specific let-7 loci.

Although at present a comprehensive picture of the regulatory function of the let-7 miRNA family cannot be drawn, it is tempting to speculate on possible roles for this miRNA family in a complex network of interacting, proproliferative/proapoptotic factors. Mounting evidence shows that the expression of the *RAS *oncogene is regulated by let-7, and that *RAS *is significantly over-expressed in lung tumour samples [40]. The 3'UTRs of the human *RAS *genes contain multiple let-7 complementary sites, allowing let-7 to regulate *RAS *[40]. In humans, let-7 is expressed in normal adult lung tissue but poorly expressed in case of lung cancers [40,71], which suggests that this miRNA may function also as a tumour suppressor. Moreover, it has been shown that over-expression of let-7 inhibited cell growth of a lung cancer cell line in vitro [71]. However, the mechanism by which let-7 regulates cell cycle is unknown. *c-MYC *is another important oncogene with the ability to induce both cell proliferation and apoptosis [86]. In addition, *RAS *and *c-MYC *function together – both are key components of the signaling pathway, regulated by the mitogen activated protein kinases (MAPK) [87,88]. It has been reported that induction of c-*MYC *expression correlates with induction of miR-17 cluster, consisting of six miRNAs: 17-5p, -18, -19a, -19b, -20 and -92 [31]. Moreover, *c-MYC *regulates the transcription of miRNAs from miR-17 cluster and two of them (miR-17-5p and miR-20) regulate transcription factor *E2F1 *at the translational level [52], which is also transcriptionally activated by *c-MYC *[89].

## Conclusion

A large number of potential miRNA target sites in 54.8% (80/149) of human oncogenes has been proposed and presented here based on an *in silico *analysis. Targets for different oncomirs, validation of which would be of special interest, have been presented. Moreover, two predicted interactions (*cMYC*/let7 and *BCL2*/miR-16) have been tested experimentally in a reporter gene assay, which presently seems to be the "gold-standard" method to validate miRNA::target interactions. Both let-7 and miR-16 have been shown to downregulate their target oncogenes, *c-MYC *and *BCL-2*, respectively. Given the known importance of the regulatory miRNA we tested (let-7) and the targeted oncogene (*c-MYC*) studied, the validation of a let-7/*c-MYC *interaction may be of particular interest. The miRNAs that are encoded by let-7 family are conserved between mammalian species, both at the sequence level and at their temporal expression patterns, which probably indicates their general role in gene regulation [16]. The *c-MYC *transcription factor, on the other hand, is one of the most potent and frequently deregulated oncoproteins in human cancers [90,91], therefore its deregulation is regarded as a hallmark of many cancers (reviewed in [92]).

## Methods

### Retrieving the Homo sapiens UTRs sequences

The UTR sequences were retrieved and collected by accurate parsing from EMBL/GenBank and "UTR Database" (UTRdb, release 15.0) [93-96]. The whole human 3'UTR dataset was provided as a multi-GenBank file. The current GeneBank format was converted to a FASTA file using BioPerl modules for biological database parsing.

### Extraction of the oncogenes

For the gathering of all oncogenes (and also some other cancer related sequences) a Perl script was developed, for parsing the local dataset files and making the onco-collection of sequences. Entries were scanned and collected by description (definition and keywords fields) from the original GenBank entries. The local database of oncogenes was extended by some sequences which were obtained from EMBL nucleotide sequence database. The final set of sequences, containing 146 entries, was structured as a multi-FASTA format file.

### miRNA binding site analysis and 2D shape filtering of RNA duplexes

All oncogene sequences were scanned with the core algorithm of *microInspector *[97]. Using the program as command line script, the software was able to process whole sets of sequences and analyse them for all possible human miRNA binding sites. The folding temperature was set to 37°C. We used a cut-off value -18 kcal/mol. For the miRNAs database we selected all *Homo sapiens *sequences from the "miRNA Registry" Release 7.1 [98].

**Figure 5:**
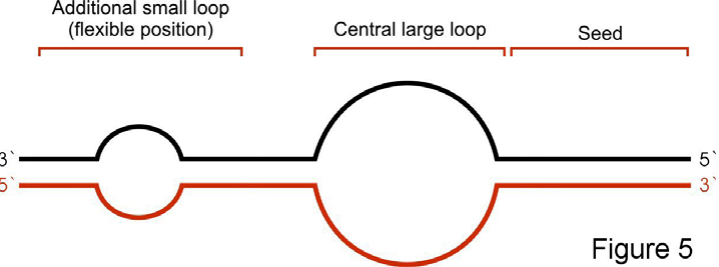
**Preferred structure for the external filter**. Outline of the miRNA-target structure selected by the external filter. The structure selected by the filter should have full complementarity in the 5'miRNA-end, optionally a central loop/bulge and good 3' pairing with up to three mismatches.

The outcome results were subjected to further examination by considering the suggested properties of animal miRNA binding sites [59] using an external Perl filter. The minimal pairing in the seed was set as 6 nucleotides, independently of nucleotide at position one. Following the main central loop/bulge (which was considered optional) the filter allowed structures having full complementarity or only few (up to 3) unpaired nucleotides at the 3' end (Fig. 5).

### Vectors and DNA constructs

To generate reporter vectors bearing miRNA-binding sites, a mammalian expression vector phRL-TK (Promega, Madison, US) carrying the *Renilla *luciferase gene (hR*luc*) as a reporter was used. It was modified in such a way to protect from re-ligation, thus simplifying the cloning of short sequences. Double restriction sites for *Bst*EII enzyme were inserted downstream from the stop codon of the luciferase gene. Specific oligonucleotides having *Bst*EII ends and containing binding sites (single or triple repeats) for the analysed microRNAs: *c-MYC *b.s. for let-7c and *BCL-2 *b.s. for miR-16 were generated (Metabion, Martinsried, Germany). The appropriate oligos were then annealed by boiling and gradually cooling down, phosphorylated and cloned into the modified phRL-TK-*Bst*EII vector at the *Bst*EII sites downstream of hR*luc *gene (Fig. 2). For all sensor constructs three types of cassettes were prepared and studied side by side: wild type (WT), carrying mutations (MUT) and perfect match (PM). In addition, to check whether the potential miRNA binding sites function in the context of the entire 3'UTR, the whole *c-MYC *3'UTR was cloned into the same luciferase vector (at *Xba *I – *Not *I restriction sites) using the following primers: Fw-5'ACTCTAGATGCGTAAGGAAAAGTAAGGA and Rev-5'AAGGCCGCATGATATATTTGCCAGTTATT. Moreover, point mutations in the potential let-7c binding site were inserted in the same positions as for the construct carrying let-7c b.s only (seed region, pos. 3^rd ^and 5^th^; compare Fig. 1B), using QuikChange Multi Site-Directed Mutagenesis Kit (Stratagene, La Jolla, CA) and primer sequence: 5'-TAGCCATAATGTAAACTG**A**C**C**CAAATTGGACTTTGGGCA. All constructs were verified by sequencing. Additionally, anti-let-7c (Dharmacon, Chicago, USA) was used to inhibit the expression of let-7c. The sequences used in our studies are listed in the Additional files (Additional file 4). Positions of mutations in the MUT constructs are indicated in bold letters.

### Transfection assay

Human HeLa 229 cell line (LGC Promochem, ATCC Number: CCL-2.1) was grown in Dulbecco's Modified Eagle's Medium (DMEM) with 10% fetal bovine serum (FBS) and gentamycine (50 μg/ml) at 37°C, in a humified atmosphere of 5% CO_2_. The cells were transfected in the 12-well plates in serum-free DMEM by using Lipofectamine 2000 (Invitrogen, Carlsbad, California) or siIMPORTER, siRNA and Plasmid DNA Transfection Reagent (Upstate, Charlottesville, USA), according to manufacturers' instructions. For each transfection experiment, 100 ng of appropriate sensor construct and 500 ng of normalization vector pCMVβ (Clontech, Mountain View, USA) expressing β-galactosidase were used in order to obtain optimal results. β-gal vector was used as a control for transfection efficiency. HeLa cells were also transfected with the phRL-TK-*BstE*II vector lacking miRNA-binding sites to demonstrate the standard luciferase activity. Cells were harvested 48 h after transfection and assayed both for luciferase activity and β-gal expression. The luciferase activity was measured using *Renilla *Luciferase Assay System (Promega, Madison, USA) with a FB 12 Luminometer (Berthold Detection Systems, USA). For the inhibition of endogenous let-7c miRNA in HeLa cells the transfection of anti-let-7c oligo (Dharmacon) at a final concentratration of 50 nM was performed, using siIMPORTER reagent (Upstate) according to manufacturers instructions. Cells were harvested 48 h after transfection and used for northern and western analyses. Inhibition of endogenous let-7c in order to restore luciferase activity of sensor constructs (Additional file 3) was achieved using the concentration of antisense let-7c oligo, as indicated in the figure. To provide an equal amount of transfected DNA the GAPAS oligo (GTGGATATTGTTGCCATCA), reported not to target any cellular miRNA, was used [66].

Figures for the tranfection assays were made by summarizing all repeats for the particular construct. Values for error bars were calculated using a formula for standard error of the mean, which is the standard deviation of the sampling distribution of the mean. The formula for the standard error of the mean is: σM = σ/√N, where σ is the standard deviation of the original distribution and N is the sample size (the repetition number).

### Northern and Western blot analyses

Total RNA was extracted from HeLa cells using Trizol Reagent (Invitrogen). Northern blot was performed as described before [99]. For probing, DNA fragments corresponding to *c-MYC *3'UTR and 18S rRNA (a loading control) were labelled by random primed incorporation of [^32^-P] dATP and dCTP (RadPrime DNA Labelling System, Invitrogen).

For the Western analysis total protein extracts from HeLa cells were used. 48 h after transfection cells were lysed with EBC buffer (50 mM Tris pH-8, 170 mM NaCl, 0.5 % NP 40, 50 mM NaF) supplemented with protease inhibitors. 30 μg of protein lysate was separated by 10% SDS-PAGE and transferred to PROTRAN nitrocellulose membrane (Schleicher & Schuell) in 25 mM Tris, 200 mM glycine, 20% methanol, at 30 V overnight at 4°C. After blocking nonspecific binding sites with 5 % nonfat milk in PBS/Tween, the membrane was incubated for 2 h at RT with primary antibody anti-cMYC (monoclonal, diluted 1:200) and then for 1 h with the secondary anti-mouse antibody (alkaline peroxidase-conjugate, diluted 1:10000). After washing in PBS/Tween, the detection was performed using SuperSignal WestPico Trial Kit (Pierce, Rockford, IL, USA)

## Abbreviations

miRNA: microRNA, b.s: miRNA binding site; *BCL-2*: B-cell leukemia/lymphoma 2; *c-MYC*: v-myc myelocytomatosis viral oncogene homolog (avian)

## Note added in proof

During the review process, work by Kumar et al. (doi: 10.1038/ng2003; 2007) was published showing that global repression of miRNA maturation promotes cellular transformation and tumorigenesis. They correlate this processes with the lack of let7 regulation on the *RAS *and *cMYC *genes.

## Competing interests

The author(s) declare that there are no competing interests.

## Authors' contributions

EK carried out the experimental validation of predicted miRNA interactions and drafted the manuscript. VB performed the *in silico *analysis and contributed in the preparation of the manuscript. KS, KO and VR participated in the experimental and bioinformatic approaches of the work, respectively. MT conceived the study. KK supervised and coordinated the project and contributed in the preparation of the manuscript. All authors read and approved the final manuscript.

## Supplementary Material

Additional file 1 Full list of predicted miRNA/oncogene interactions. Click here for file

Additional file 2 Predicted interactions of different human and murine *MYC *mRNAs with miRNAs of the let-7 family. GeneBank accession numbers and the positions of the binding motifs (all within the 3'UTR) and the calculated free energies are indicated. Click here for file

Additional file 3 Luciferase activity following transfections of reporter constructs with let-7c microRNA inhibitor. Constructs used for the validation of let7c/*c-MYC *interaction (WT, MUT and PM) were co-transfected into HeLa cells together with anti-let-7c inhibitor at two different concentrations, as indicated in the figure. Results are presented in % (100% – luciferase vector with no binding site). To provide an equal amount of transfected DNA the GAPAS oligo, not targeting any cellular miRNA, was used [66]. Click here for file

Additional file 4 Sequences of oligos used to create sensor constructs carrying *c-MYC *b.s. for let-7c and *BCL-2 *b.s. for miR-16. Three types of cassettes (WT, MUT and PM) were constructed as described in the text. Positions of mutations in the MUT constructs are indicated in bold, underlined letters. Abbreviations 1 bs and 3 bs stand for single and triple binding site, respectively. Sense or antisense strand of the oligo is indicated by letters A and B. Click here for file
