# Supplementary figures and images for: Prediction and preliminary validation of oncogene regulation by miRNAs
Source: BMC Mol Biol. 2007 Sep 18;8:79. doi: 10.1186/1471-2199-8-79 (PMC2096627; doi:10.1186/1471-2199-8-79)

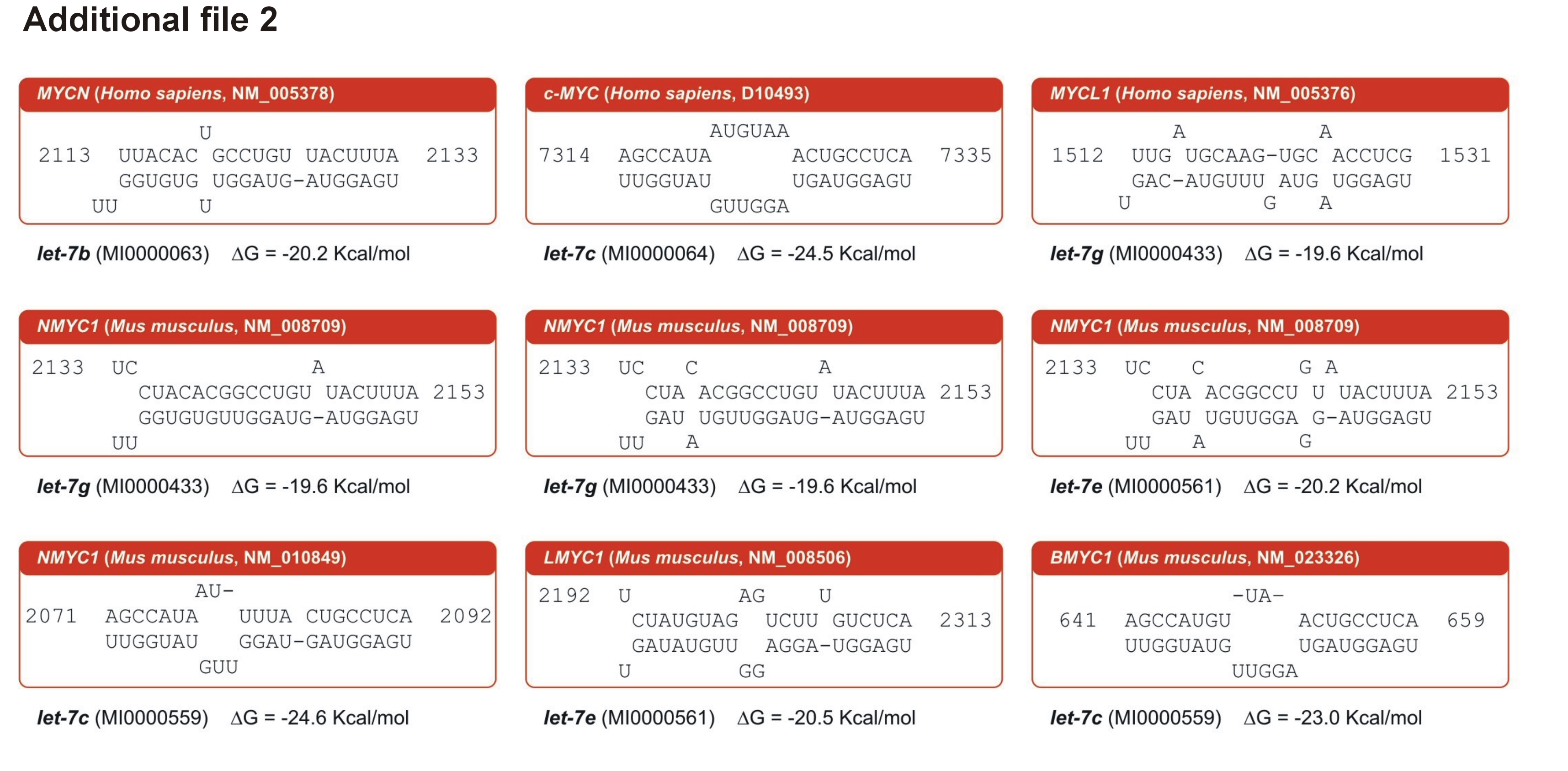

Supplement: Additional file 2 — Predicted interactions of different human and murine MYC mRNAs with miRNAs of the let-7 family. GeneBank accession numbers and the positions of the binding motifs (all within the 3'UTR) and the calculated free energies are indicated. [file 1471-2199-8-79-S2.jpeg]

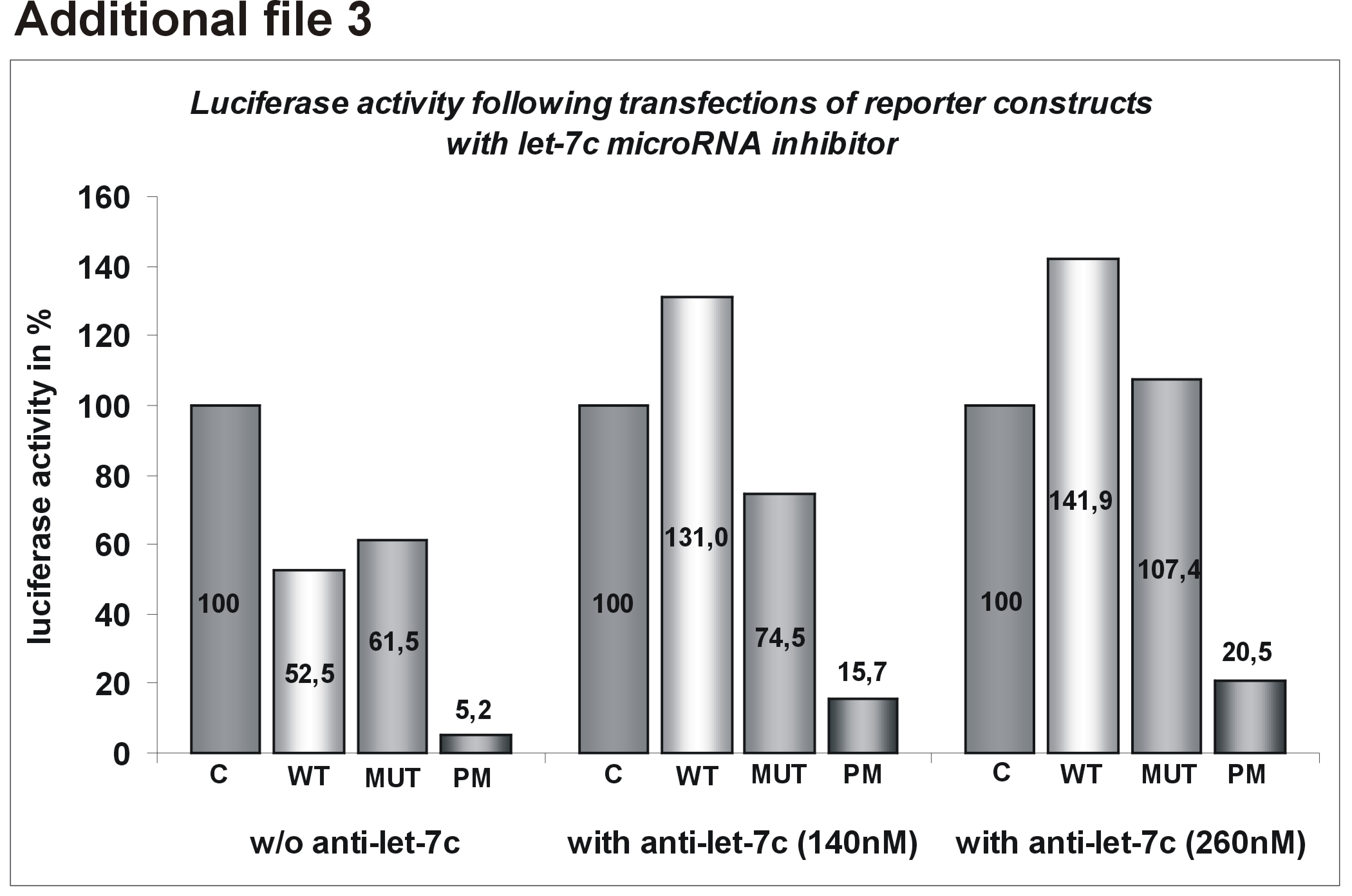

Supplement: Additional file 3 — Luciferase activity following transfections of reporter constructs with let-7c microRNA inhibitor. Constructs used for the validation of let7c/c-MYC interaction (WT, MUT and PM) were co-transfected into HeLa cells together with anti-let-7c inhibitor at two different concentrations, as indicated in the figure. Results are presented in % (100% – luciferase vector with no binding site). To provide an equal amount of transfected DNA the GAPAS oligo, not targeting any cellular miRNA, was used [66]. [file 1471-2199-8-79-S3.jpeg]
